# ACSL4 predicts rapid kidney function decline in patients with diabetic kidney disease

**DOI:** 10.3389/fendo.2025.1499555

**Published:** 2025-03-13

**Authors:** Rui Shen, Xin Yu, Caifeng Shi, Yi Fang, Chunsun Dai, Yang Zhou

**Affiliations:** Center for Kidney Disease, The Second Affiliated Hospital of Nanjing Medical University, Nanjing, China

**Keywords:** ACSL4, diabetic kidney disease, eGFR slope, proteinuria, biopsy

## Abstract

**Background:**

Ferroptosis of kidney tubular epithelial cells contributes to the pathogenesis of diabetic kidney disease (DKD). An increase in the enzyme long-chain fatty acid CoA ligase 4 (ACSL4) favors ferroptosis. However, the association between ACSL4 in renal tubules and kidney outcomes of patients with DKD is unknown.

**Methods:**

To investigate the predictive property of ACSL4 in rapid kidney function decline in patients with DKD, a retrospective cohort of 72 biopsy-proven DKD patients were enrolled and followed up for a median of 23 months. Tubular expression levels of ACSL4 in the renal biopsy specimens from 72 DKD patients and 12 control subjects were measured using immunohistochemistry staining. The associations between the ACSL4 level and clinical characteristics as well as rapid kidney function decline defined as an estimated glomerular filtration rate (eGFR) slope ≤ -5 ml/min/1.73m^2^/year were analyzed.

**Results:**

ACSL4 was mainly expressed in tubular epithelial cells. The tubular ACSL4 expression levels in the DKD patients were significantly higher than those in the control subjects. ACSL4 was positively correlated with proteinuria and negatively correlated with albumin and hemoglobin at the time of the renal biopsy. During the follow-up time period, the median eGFR slope of these DKD patients was -2.30 ml/min/1.73m^2^/year. ACSL4 was negatively correlated with the eGFR slope. The top tertile of baseline ACSL4 was found to identify the subjects with DKD who were at high risk for rapid kidney function decline and a similar significant relationship was found using ACSL4 levels as a continuous variable.

**Conclusions:**

ACSL4 was associated with a rapid progression of DKD and may serve as a novel pathological biomarker.

## Introduction

Diabetic kidney disease (DKD) occurs in up to 20%–40% of patients with diabetes and has become the leading cause of chronic kidney disease (CKD), renal failure, and premature death (1). The classic clinical phenotype of DKD consists of persistently increased albuminuria and a progressive decline in glomerular filtration rate (GFR) (2). However, alternative trajectories of kidney function in patients with diabetes have been reported and characterized by a rapid decline in GFR, regression in albuminuria, or absence of albuminuria or proteinuria (3). The annual median estimated GFR (eGFR) varied from -4.0 to -1.5 ml/min/1.73m^2^ in patients with diabetes (4). Those with an eGFR slope ≤ -5 ml/min/1.73m^2^/year are termed as having rapid kidney function decline (5). A higher rate of eGFR decline was considered to be associated with a higher risk of subsequent renal failure and cardiovascular comorbidities (6).

Observational studies suggested that albuminuria or macroalbuminuria may not indicate a rapid decline in eGFR (7). Although kidney biopsies are not typically required for the diagnosis of DKD, a strong relationship has been observed between histological findings and clinical presentations and progressions (8). A loss of kidney tubules correlates with the decline in eGFR in advanced DKD (9). Ferroptosis is an iron-dependent regulated cell death that is driven by robust cellular lipid oxidation and is inhibited by thiol-dependent peroxidase (10). Tubular cells have active iron reabsorption, a high lipid metabolic rate, and concentrated redox-active compound perturbations, which constitute the main pillars of ferroptosis (11, 12). Our previous data revealed beneficial effects on kidney tubular protection in diabetes by suppressing ferroptosis (13). It was reported that upregulation of the pro-ferroptotic protein long-chain fatty acid CoA ligase 4 (ACSL4) appeared in tubular cells in various human kidney diseases (14). The tubular expression of ACSL4 has been observed in mouse models and patients with DKD, and it was negatively correlated with the patients’ eGFR (15). However, the previous study group consisted of various etiologies and lacked specificity in DKD. Moreover, the association between the tubular expression of ACSL4 and kidney outcomes of patients with DKD is unknown.

Therefore, in this retrospective cohort study, we enrolled patients with biopsy-proven DKD to investigate the association between the tubular expression of ACSL4 and a rapid decline in kidney function.

## Materials and methods

### Study patients

**Figure 1:**
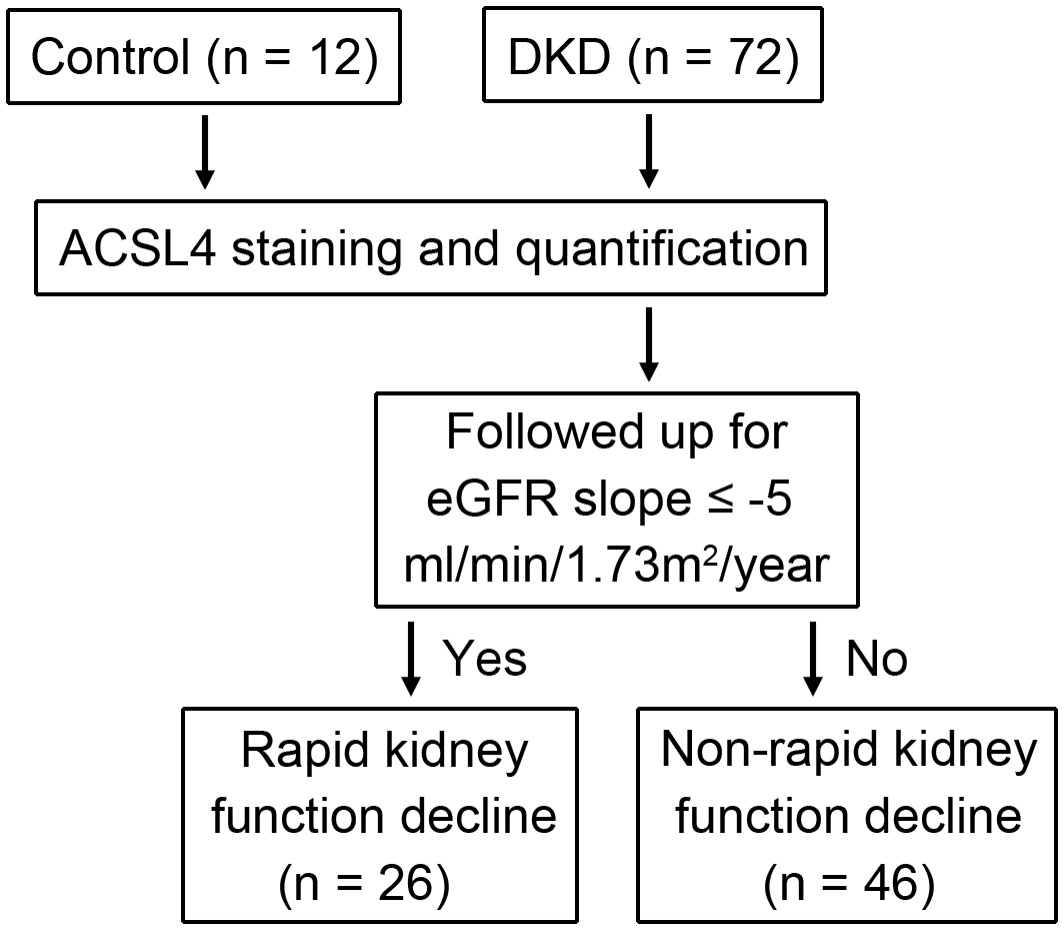
Schematic of the analytical strategy employed in this study.

We enrolled patients with type 2 diabetes confirmed according to American Diabetes Association 2018 criteria and with kidney biopsy-proved DKD according to Renal Pathology Society criteria (16) from The Second Affiliated Hospital of Nanjing Medical University. A kidney biopsy for patients with diabetes and CKD was performed due to the presence of at least one of the following indications (17): rapid onset of proteinuria, presence of active urinary sediment (e.g., hematuria), rapid decrease in renal function, absence of retinopathy, resistant hypertension, and suspicion of other nephropathies secondary to systemic disease. Patients coexisting with non-diabetic kidney disease (NDKD) were excluded. In addition, patients with acute kidney injury, acute inflammatory diseases, systemic diseases, malignant neoplasm, endocrine disorders other than diabetes, or who had received kidney replacement therapy at recruitment were excluded. Kidney specimens with biopsy-proven mild mesangial hyperplasia and without tubulointerstitial damage from 12 patients without diabetes or secondary glomerulonephritis were recruited as controls (**Figure 1**).

### Laboratory examination and outcomes

At the time of the renal biopsy, age, sex, diabetes history, systolic and diastolic blood pressure, hemoglobin, albumin, fast blood glucose level, hemoglobin A1c (HbA1c), total cholesterol, triglyceride, uric acid, creatinine, and proteinuria were systemically recorded. eGFR was calculated using the Chronic Kidney Disease Epidemiology Collaboration equation (CKD-EPI equation, http://www.nkdep.nih.gov) (18). The median eGFR slope of the DKD patients was calculated as the average change from baseline to at least two time points every year using the least square method (19). The outcome was rapid kidney function decline defined as an eGFR slope ≤ -5 ml/min/1.73m^2^/year (3).

### ACSL4 immunohistochemistry staining

Formalin-fixed paraffin-embedded kidney tissue sections from the controls (n = 12) and DKD patients (n = 72) were deparaffinized and blockaded with bovine albumin. After heat-induced retrieval for 15 min at 95°C in pH 9.0 Tris/EDTA buffer, tissue sections were stained with anti-ACSL4 antibody (ab155282, Abcam, Cambridge, MA, US). Images were obtained with an Eclipse 80i microscope (Nikon) equipped with a digital camera (DS-Ri1, Nikon, Shanghai, China). Ten random images for each section under 400× magnification in a blind fashion were taken for quantification, demonstrated by integrated optical density per area (IOD/area), using Image-Pro Plus software (Media Cybernetics, Bethesda, MD).

### Statistics analysis

The Shapiro–Wilk test was used to determine the distribution of the variables. Continuous data were presented as mean ± SD or median and interquartile range (IQR) as appropriate. Categorical data were described by absolute frequencies and percentages. Continuous clinical data were compared appropriately using Student’s t-test or the Mann–Whitney U test. The Pearson or Spearmen correlation analysis was performed to evaluate the association between the log2 transformed IOD/area of ACSL4 and clinical parameters. The Kaplan–Meier analysis was used to assess the association between the IOD/area of ACSL4 and rapid kidney function decline. The Cox proportional hazard regression was employed to evaluate the association between ACSL4 and rapid kidney function decline after adjusting for sex, baseline age, blood pressure, HbA1c, eGFR, and proteinuria. The analysis was performed with the SPSS statistical software package V.24.0 (IBM). A *p*-value less than 0.05 was considered statistically significant.

## Results

### Increased expression of ACSL4 in kidney tubular epithelial cells of patients with DKD

Expressions of ACSL4 in kidney tissue were examined using renal biopsy specimens from controls and DKD patients (**Figure 1**). The detailed clinical characteristics are listed in **Table 1**. Among the enrolled 72 DKD patients, 21 were female, with an age of 56 years (50, 63) and a duration of diabetes of 96 months (36, 180) at the time of the renal biopsy. The HbA1c level of these patients was 7.50% (6.60%, 8.55%). The proteinuria and eGFR of the patients were 3.46 g/24h (0.84, 6.64) and 56.19 ml/min/1.73m^2^(42.52, 83.01), respectively.

**Table 1:** Baseline clinical characteristics of the control subjects and participants with diabetic kidney disease.

Characteristic	Controls (n = 12)	DKD patients (n = 72)
Female sex, n (%)	9 (75%)	21 (29.17%)
Index age, years	48.0 (37.5, 58.5)	56.0 (50.0, 63.0)
Duration of diabetes, months	/	96 (36, 180)
SBP, mmHg	123.08 ± 26.62	152.74 ± 22.54
DBP, mmHg	80.75 ± 20.65	87.72 ± 12.11
Hemoglobin, g/L	135.30 ± 7.85	120.42 ± 26.96
Albumin, g/L	44.44 ± 3.61	36.10 ± 8.58
FBG, mmol/L	4.58 (4.08, 5.60)	7.74 (6.10, 10.77)
HbA1c, %	5.40 (5.25, 5.58)	7.50 (6.60, 8.55)
Total cholesterol, mmol/L	4.97 (4.30, 5.39)	5.35 (3.90, 7.00)
Triglyceride, mmol/L	1.08 (0.86, 3.69)	1.87 (1.18, 3.04)
Uric acid, μmol/L	304.44 ± 92.76	362.29 ± 113.09
Creatinine, μmol/L	71.20 (52.95, 78.85)	112.45 (83.78, 145.55)
Proteinuria, g/24h	0.17 (0.13, 0.21)	3.46 (0.84, 6.64)
eGFR, ml/min/1.73m^2^	105.48 (85.30, 117.77)	56.19 (42.52, 83.01)

DBP, diastolic blood pressure; DKD, diabetic kidney disease; eGFR, estimated glomerular filtration rate; FBG, fast blood glucose; HbA1c, hemoglobin A1c; SBP, systolic blood pressure.

**Figure 2:**
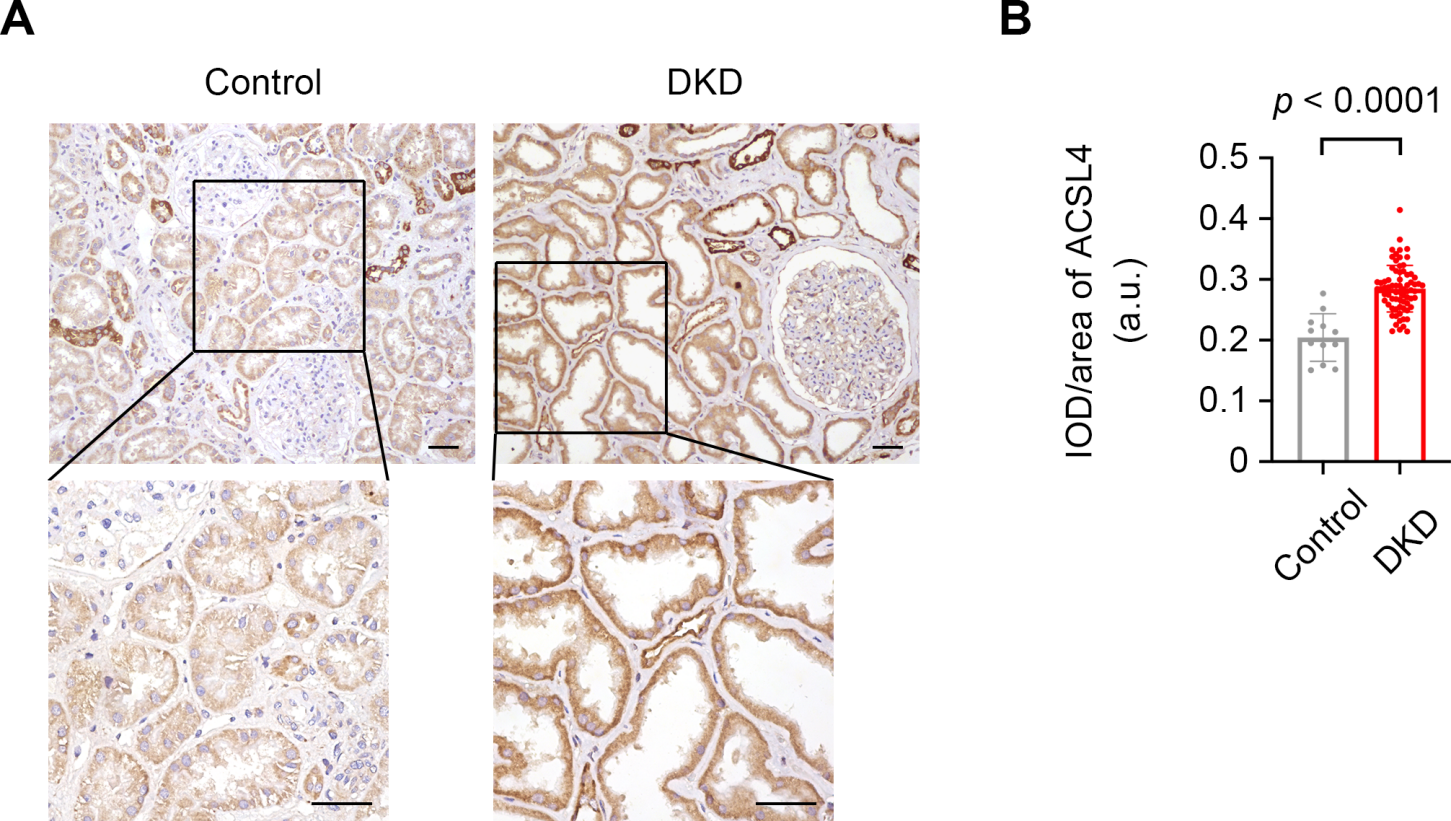
The increased expression levels of ACSL4 in kidney tissue specimens from control and DKD subjects. **(A)** Representative photographs of kidney tissue specimens from the controls and DKD subjects immunostained with ACSL4. Scale bar = 50 μm for both the larger image and the magnified image. **(B)** Quantification of the expression levels of ACSL4 in kidney tubules from controls (n = 12) and DKD subjects (n = 72) demonstrated by integrated optical density per area (IOD/area). Data are presented as mean ± SD.

Immunostaining of the kidney tissue section indicated that ACSL4 was mainly expressed in tubular epithelial cells (**Figure 2A**). The tubular ACSL4 expression levels evaluated by IOD/area were significantly higher in the DKD patients than in the control subjects (0.28 ± 0.04 vs. 0.20 ± 0.04, *p* < 0.0001) (**Figure 2B**).

### Association between tubular ACSL4 levels and clinical characteristics of patients with DKD

The correlation analysis showed that tubular expression levels of ACSL4 were positively correlated with proteinuria (*r* = 0.28, *p* = 0.018) and negatively correlated with albumin (*r* = -0.32, *p* = 0.007), and hemoglobin (*r* = -0.26, *p* = 0.027) at the time of the renal biopsy (**Table 2**). No significant association was found between tubular ACSL4 levels and age, sex, duration of diabetes, blood pressure, blood glucose, creatinine, and eGFR at the time of the renal biopsy.

**Table 2:** Correlations between tubular ACSL4 expression levels and clinical characteristics of patients with diabetic kidney disease.

Characteristic	*r* value	*p* value
Albumin	-0.32	0.007
Proteinuria	0.28	0.018
Hemoglobin	-0.26	0.027
eGFR slope	-0.26	0.027
Male sex	0.01	0.966
Index age	-0.05	0.651
Duration of diabetes	-0.03	0.838
SBP	0.08	0.501
DBP	-0.01	0.931
FBG	-0.03	0.823
HbA1c	-0.16	0.215
Total cholesterol	0.05	0.673
Triglyceride	-0.02	0.869
Uric acid	0.05	0.692
Creatinine	0.14	0.259
eGFR	-0.17	0.154

DBP, diastolic blood pressure; eGFR, estimated glomerular filtration rate; FBG, fast blood glucose; HbA1c, hemoglobin A1c; SBP, systolic blood pressure.

During the follow-up of 23.00 months (13.25, 44.50), the eGFR slope of these DKD patients was -2.30 ml/min/1.73m^2^/year (-8.11, -0.92), indicating a decline in the kidney function of the patients with DKD. The tubular expression levels of ACSL4 were negatively correlated with the eGFR slope (*r* = -0.26, *p* = 0.027) (**Table 2**).

### Association between tubular ACSL4 levels and rapid kidney function decline in DKD

A total of 26 (36.11%) DKD patients progressed rapidly with an eGFR slope at -12.50 ml/min/1.73m^2^/year (-22.05, -6.97) during the follow-up period. Furthermore, the eGFR slope of the DKD patients with stable kidney function was -1.26 ml/min/1.73m^2^/year (-2.23, -0.28) (*p* < 0.001 compared to those with rapid kidney function decline). The baseline clinical characteristics of DKD patients with stable or rapid kidney function decline are listed in **Table 3**. As compared to DKD patients with stable kidney function during the follow-up period, tubular expression levels of ACSL4 at baseline were significantly higher in the DKD patients with rapid kidney function decline (0.27 ± 0.03 vs. 0.30 ± 0.04, *p* = 0.002), indicating it could be an early marker of future kidney function decline.

**Table 3:** Baseline clinical characteristics of the DKD patients with stable kidney function or rapid kidney function decline.

Characteristic	Stable kidney function (n = 46)	Rapid kidney function decline (n = 26)	*p* value
Female sex, n (%)	13 (28.26%)	8 (30.77%)	0.822
Index age, years	55.50 (50.75,64.00)	57.5 (47.25, 63.00)	0.974
Duration of diabetes, months	96.00 (24.00,204.00)	96.00 (36.00, 120.00)	0.855
SBP, mmHg	152.22 ± 24.34	153.65 ± 19.38	0.797
DBP, mmHg	85.00 (79.00, 95.25)	86.50 (80.00, 96.25)	0.397
Hemoglobin, g/L	119.65 ± 26.66	121.77 ± 27.97	0.668
Albumin, g/L	36.82 ± 7.86	34.85 ± 9.76	0.353
FBG, mmol/L	7.69 (6.08, 11.60)	7.74 (6.32, 9.95)	0.733
HbA1c, %	7.60 (6.70, 8.70)	7.10 (6.45, 8.30)	0.256
Total cholesterol, mmol/L	5.34 (3.77,7.06)	5.38 (4.11,6.66)	0.810
Triglyceride, mmol/L	1.81 (1.18, 3.24)	1.90 (1.24, 3.01)	0.952
Uric acid, μmol/L	357.50 ± 97.08	370.92 ± 139.18	0.638
Creatinine, μmol/L	118.10 (90.33,166.08)	98.25 (75.68, 132.23)	0.102
Proteinuria, g/24h	2.84 (0.57, 6.76)	3.48 (1.22, 6.49)	0.470
eGFR, ml/min/1.73m^2^	60.50 ± 31.40	72.26 ± 33.98	0.143
IOD/area of ACSL4	0.27 ± 0.03	0.30 ± 0.04	0.002

DBP, diastolic blood pressure; DKD, diabetic kidney disease; eGFR, estimated glomerular filtration rate; FBG, fast blood glucose; HbA1c, hemoglobin A1c; IOD, integrated optical density; SBP, systolic blood pressure.

**Figure 3:**
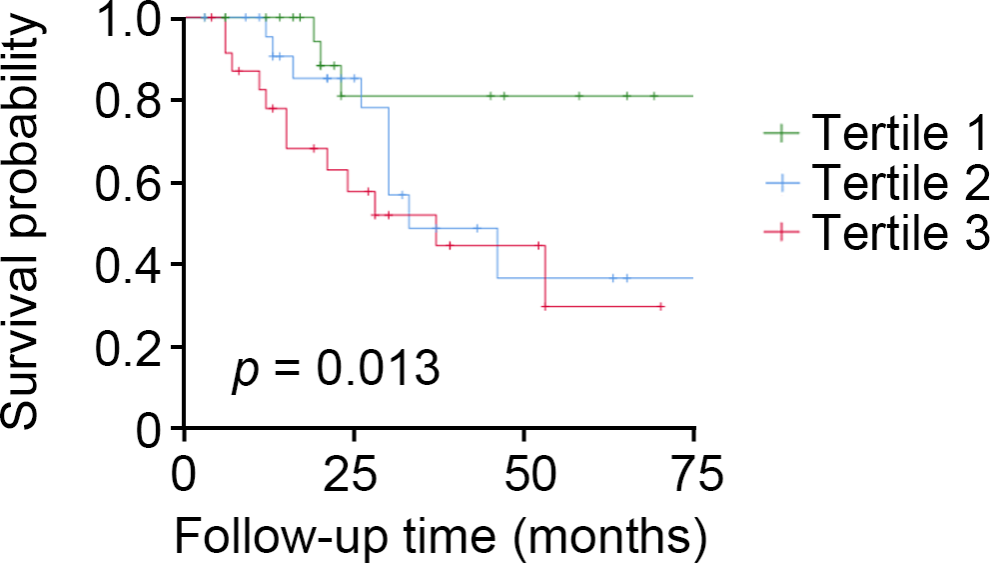
High tubular expression levels of ACSL4 identify patients with DKD who are at high risk of rapid kidney function decline. Participants with DKD (n = 72) who had tubular expression levels of ACSL4 analyzed were followed for 23 months. Kaplan–Meier analysis shows that participants with the top tertile had a higher risk of rapid kidney function decline.

The Kaplan–Meier and multivariate Cox proportional hazard regression model analyses showed that the top tertile of baseline tubular expression level of ACSL4 was found to identify the subjects with DKD who were at high risk for rapid kidney function decline [adjusted HR 7.92, 95% CI (1.44, 43.44), as compared to lowest tertile] (**Figure 3**) and a similar significant relationship was found using tubular ACSL4 levels as a continuous variable [adjusted HR 1.76, 95% CI (1.25, 2.48)] (**Table 4**).

**Table 4:** Association of baseline tubular ACSL4 expression levels with risk for rapid progression of diabetic kidney disease in the study cohort.

ACSL4	Total (n = 72)	
	HR (95% CI)	*p*-value
1-SD increment	1.76 (1.25, 2.48)	0.001
Tertile 2 vs. Tertile 1	5.32 (1.01, 27.91)	0.048
Tertile 3 vs. Tertile 1	7.92 (1.44, 43.44)	0.017

Multivariate Cox proportional hazard regression models were adjusted for sex, baseline age, blood pressure, HbA1c, eGFR, and proteinuria. Tubular ACSL4 expression levels were modeled as both a continuous variable (1-SD increment in log_2_-transformed IOD/area of ACSL4) and a categorical variable (the lowest tertile as reference).

## Discussion

In this study, immunostaining showed that ACSL4 was mainly expressed in tubular epithelial cells in kidney biopsy specimens from human subjects. The increased expression levels of ACSL4 in kidney tubules from patients with DKD were further positively correlated with proteinuria at the time of the renal biopsy. Multivariate Cox proportional hazard regression models adjusted for sex, baseline age, blood pressure, HbA1c, eGFR, and proteinuria showed that patients with a high baseline tubular expression level of ACSL4 were at high risk for rapid kidney function decline when ACSL4 was modeled as both a continuous variable (1-SD increment in log2-transformed IOD/area of tubular ACSL4) and a categorical variable (the lowest tertile as reference).

Common risk factors for the rapid progression of DKD include high blood glucose, high blood pressure, proteinuria, and low GFR category (20). It is assumed that a rapid decline in kidney function has likely become less common since multifactorial interventions have become available in clinical settings. Notably, our previous study indicated that sodium-glucose cotransporter 2 (SGLT2) inhibitors and renin-angiotensin-aldosterone system inhibitors were not routinely used in more than 66% and 77% of the DKD patients we followed, respectively (21). Nevertheless, 26/72 (36.11%) patients with DKD progressed rapidly, as demonstrated by an eGFR slope < -5 ml/min/1.73m^2^/year. An increase in tubular expression levels of ACSL4 evaluated at the time of the renal biopsy helped identify patients with a high risk of rapid kidney function decline independent of the baseline blood glucose, blood pressure, proteinuria, and eGFR, suggesting a new approach for precision intervention. Additionally, this study may validate tubular ACSL4 as an early marker with the advantage of pre-dating the functional decline and other risk factors.

Previous studies found tubular cell ferroptosis in models of diabetes and suggested that inhibition of ferroptosis relieved the development and progression of DKD (13, 22–24). Increased ACSL4 facilitates the availability of lipid substrates and favors ferroptosis. However, the presence of ACSL4 in the control subjects does not necessarily indicate ferroptosis as ACSL4 itself is involved in lipid biosynthesis. Moreover, a deficiency of ACSL4 prevented ferroptosis but favored the occurrence of necroptosis (25). The pathologic mechanisms of increased ACSL4 in tubular epithelial cell death and progression of DKD should be investigated in further studies.

Consistent with our study, a previous study found that decreased tubular expression of glutathione peroxidase 4 (GPX4) predicted DKD progression (26). GPX4 is an intracellular antioxidant enzyme that protects cells against lipid peroxidation and therefore prohibits ferroptosis (27). In animal models or cultured cells, ferroptosis is always evaluated by ACSL4 upregulation, GPX4 reduction, ferrous iron overload, and lipid peroxidation aggregation. It is plausible that the increased expression of ACSL4 may be an appropriate pathologic biomarker for clinical applications.

Proteinuria typically indicates dysfunction in the glomerular filtration barrier, while ACLS4 expression in tubules suggests injuries of tubular cells. The relationship between proteinuria and ASCL4 may indicate an interplay between tubules and glomeruli (28) underlying the progression of DKD and led us to consider tubuloglomerular feedback mechanisms. Furthermore, significantly reduced proteinuria and mitigated glomerular hypertrophy were found by suppressing ferroptosis in a diabetic model (13). However, it was not easily explained by directly blocking adverse glomerular hemodynamics, such as with SGLT2 inhibitors. Further studies are needed.

It cannot be definitively stated that a person with diabetes and CKD has DKD unless the person has a kidney biopsy; however, in most cases, a kidney biopsy was not recommended as other possible diagnoses would not change treatment (29). A kidney biopsy is performed due to the presence of at least one of the following indications (17): rapid onset of proteinuria, presence of active urinary sediment (e.g., hematuria), rapid decrease in renal function, absence of retinopathy, resistant hypertension, and suspicion of other nephropathies secondary to systemic disease. Therefore, kidney biopsy samples from patients in the DKD stage I or IV were scant due to the absence of indications or the presence of contraindications. We observed a positive correlation between tubular ACSL4 and proteinuria, which probably indicates a tubuloglomerular interplay as we mentioned above; however, the relationship between tubular expression of ACSL4 and diabetic nephropathy defined by the Renal Pathology Society as well as proteinuria, which both primarily describe glomerular lesions, remains obscure.

The current study has some strengths. We identified tubular expression levels of ACSL4 as a novel biomarker for rapid kidney function decline in subjects with DKD. There were some limitations of our study. The sample size was limited and most of the patients enrolled were at the DKD stages II and III (16). The role of ACSL4 in the progression of DKD remains obscure and whether ACSL4 contributes to CKD with other etiologies needs further investigation.

In conclusion, this study showed that a significantly increased expression of ACSL4 in kidney tubules was associated with more severe proteinuria and a higher risk of rapid kidney function decline as evaluated by eGFR slope in patients with DKD.

## Data Availability

The raw data supporting the conclusions of this article will be made available by the authors, without undue reservation.
